# Risk Factors and Nomogram for Predicting Relapse Risk in Pediatric Neuromyelitis Optica Spectrum Disorders

**DOI:** 10.3389/fimmu.2022.765839

**Published:** 2022-02-18

**Authors:** Shanchao Zhang, Shan Qiao, Haiyun Li, Ranran Zhang, Meiling Wang, Tao Han, Xuewu Liu, Yunshan Wang

**Affiliations:** ^1^ Medical Research and Laboratory Diagnostic Center, Jinan Central Hospital, Cheeloo College of Medicine, Shandong University, Jinan, China; ^2^ School of Medicine, Cheeloo College of Medicine, Shandong University, Jinan, China; ^3^ Department of Neurology, The First Affiliated Hospital of Shandong First Medical University & Shandong Provincial Qianfoshan Hospital, Jinan, China; ^4^ Department of Medical Genetics, School of Basic Medical Sciences, Cheeloo College of Medicine, Shandong University, Jinan, China; ^5^ Department of Neurology, Qilu Hospital, Cheeloo College of Medicine, Shandong University, Jinan, China; ^6^ Department of Neurology, Binzhou Medical University Hospital, Binzhou, China; ^7^ Department of Neurology, Shandong Provincial Hospital, Shandong University, Jinan, China; ^8^ Institute of Epilepsy, Shandong University, Jinan, China; ^9^ Basic Medical Research Center, Jinan Central Hospital, Cheeloo College of Medicine, Shandong University, Jinan, China

**Keywords:** aquaporin-4, myelin oligodendrocyte glycoprotein, neuromyelitis optica spectrum disorders, relapse prediction, pediatric patient

## Abstract

**Background:**

Neuromyelitis optica spectrum disorders (NMOSDs) are attack-relapsing autoimmune inflammatory diseases of the central nervous system, which are characterized by the presence of serological aquaporin-4 (AQP4) antibody. However, this disorder is uncommon in children, and AQP4 antibody was often found to be seronegative. However, some pediatric patients diagnosed with NMOSDs were tested to be positive for myelin oligodendrocyte glycoprotein (MOG) antibody. The previous investigations of pediatric NMOSDs were usually focused on the clinical presentation, treatment responses, and long-term prognoses, but little is known about the risk factors predicting NMOSD relapse attacks in a shorter time, especially, for Chinese children.

**Methods:**

We retrospectively identified 64 Chinese pediatric patients, including 39 positive for AQP4 antibody, 12 positive for MOG antibody, and the rest negative for AQP4 and MOG antibodies. Independent risk factors predicting relapse in 1-year follow-up were extracted by multivariate regression analysis to establish a risk score model, its performance evaluation was analyzed using receiver operating characteristic (ROC) curve, and the independent risk factors related to relapse manifestation were also explored through multivariate logistic analysis. A nomogram was generated to assess relapse attacks in 1-year follow-up. Thirty-five patients from 3 other centers formed an external cohort to validate this nomogram.

**Results:**

Four independent relapsed factors included discharge Expanded Disability Status Scale (EDSS) (p = 0.017), mixed-lesion onset (p = 0.010), counts (≧1) of concomitant autoantibodies (p = 0.015), and maintenance therapy (tapering steroid with mycophenolate mofetil (MMF), p = 0.009; tapering steroid with acetazolamide (AZA), p = 0.045; and tapering steroid only, p = 0.025). The risk score modeled with these four factors was correlated with the likelihood of relapse in the primary cohort (AUC of 0.912) and the validation cohort (AUC of 0.846). Also, our nomogram exhibited accurate relapse estimate in the primary cohort, the validation cohort, and the whole cohort, but also in the cohorts with positive/negative AQP4 antibody, and noticeably, it performed predictive risk improvement better than other factors in the concordance index (C-index), net reclassification improvement (NRI), and integrated discrimination improvement (IDI).

**Conclusions:**

The risk score and nomogram could facilitate accurate prognosis of relapse risk in 1-year follow-up for pediatric NMOSDs and help clinicians provide personalized treatment to decrease the chance of relapse.

## Introduction

Neuromyelitis optica spectrum disorders (NMOSDs) are defined as a category of autoantibody-induced central nervous system (CNS) inflammatory diseases characterized by recurrent attacks targeting the optic nerves, spinal cord, or brain/brainstem. The presence of pathogenic aquaporin-4 (AQP4) antibody in serum, targeting water channel protein AQP4 expressed in the endfeet of astrocytes in CNS that is highly specific for NMOSDs (1), could induce the loss of astrocytic AQP4 and the myelin sheath, axonal injury, activated complement components, and inflammatory infiltration with granulocytes (2). The clinical features and abnormalities of AQP4 antibody-positive pediatric NMOSD patients are similar to those of the adult phenotype (3, 4). Fortunately, children commonly have a less severe clinical course and relapses, and disability may take longer in children than in adults (5), despite being more likely to have a visual impairment (6). Intriguingly, recent studies revealed that some children with NMOSD could acquire AQP4-negative autoimmunity (7, 8), indicating that myelin oligodendrocyte glycoprotein (MOG) antibody targeting MOG antigen on myelin sheaths of oligodendrocyte (9), mainly concomitant with CD4+ T cells and granulocyte infiltration, and complement deposition not observed on astrocytes or glia limitans (10), was detectable in some pediatric patients with AQP4 antibody-negative NMOSDs (11).

Pediatric NMOSDs commonly manifest as recurrent attacks of variable symptoms if untreated and have a high risk of permanent visual and motor deficit due to the stepwise accumulation of disability (12). Hence, the accurate prediction of relapse and the early attack-preventing treatment are crucial for clinical outcomes. Several relapse-related predictors have been reported in previous studies, for example, the short duration from disease onset to the first relapse and the high annualized relapse rate (ARR) pretreatment, and these factors may indicate a high risk of relapse in children with AQP4 antibody-positive NMOSDs (13). Furthermore, multiple onsets like acute disseminated encephalomyelitis (ADEM) plus optic neuritis (ON) or ON plus transverse myelitis (TM), etc., represent high relapse frequency in a shorter time for the children with MOG antibody-positive NMOSDs (14). As for Chinese children, NMOSDs are considered as the common type of acquired CNS demyelinating diseases (6); however, it remains elusive regarding the relapse risk factors and even the clinical course and prognosis for Chinese pediatric NMOSDs.

Our study aimed to further investigate demographics, disease onset, and treatment responses of Chinese pediatric NMOSDs and to identify independent risk factors predicting relapse attacks and relapse presentation.

## Materials and Method

### Patients and Data Collection

We included all pediatric patients (age of onset ≤18 years) from 2016 to September 2020, diagnosed by the international consensus in 2015 for NMOSDs (15). Patients who met the 2006 Wingerchuk criteria were also included based on the 2015 consensus (16). In the primary cohort, 64 children from Qilu Hospital, Cheeloo College of Medicine, Shandong University, were recruited to identify the risk factors for predicting the occurrence of relapse; and 35 children in the validation cohort from Shandong Provincial Hospital, Shandong University (n = 19), the First Affiliated Hospital of Shandong First Medical University and Shandong Provincial Qianfoshan Hospital (n = 7), and Binzhou Medical University Hospital (n = 9) were verified in terms of discrimination and clinical profit of these risk factors. Medical records were retrospectively reviewed, and the data were collected for demographic details, including clinical onset, laboratory data, cerebrospinal fluid (CSF) findings, AQP4 and MOG antibody status, and treatment management. Visual evoked potentials were collected to confirm ON if needed. Expanded Disability Status Scale (EDSS) score was collected retrospectively from available medical records and classified as a baseline before admission, pretreatment on admission, discharge, and 1-year follow-up. The data in follow-up were acquired through clinical examinations during return visits and telephone surveys. All data were excluded, as follows: 1) incomplete information or with a history of other CNS disease (e.g., multiple sclerosis, systemic lupus erythematosus, paraneoplastic tumor, and related diseases); 2) seropositive for AQP4 plus MOG antibody or unknown AQP4/MOG antibody status; and 3) exposure to other immunosuppressants (e.g., rituximab, tacrolimus, tocilizumab, and cyclophosphamide) or having more than one agent before pretreatment.

### Antibody Status

Serum and CSF AQP4/MOG antibodies were detected in all recruited pediatric patients using the fixed cell-based assay on HEK293 cells transfected with MOG and AQP4 (17, 18).

### Onset Episode

To evaluate the frequencies of different clinical symptoms at onset, we categorized the following clinical episode: isolated ON, isolated TM, isolated brain/brainstem, and mixed lesions (e.g., ON+TM, ON+brain/brainstem, TM+brain/brainstem, and ON+TM+ brain/brainstem).

### Clinical and Outcome Assessment

Relapses were defined as new or worsening neurological symptoms lasting longer than 24 h without other etiology and demonstrated by new neurological examination findings and/or new lesions on MRI (19). The clinical status was based on EDSS and visual acuity. The visual disability referred to ≤0.1 based on the standard table of vision logarithms. The concomitant autoantibodies consisted of antinuclear antibody, extractable nuclear antigen antibody, double-stranded DNA antibody, antineutrophil cytoplasmic antibody, anticardiolipin antibody, Sjögren’s syndrome A antibody, Sjögren’s syndrome B antibody, rheumatoid factor, anti-oipA antibody, thyroglobulin antibody, and thyroid peroxidase antibody, not including AQP4/MOG antibody. For acute therapy, intravenous high-dose steroid was administered with a dose of 20–30 mg/kg/day for 3–5 days, and then steroid dosage gradually tapered to oral prednisone therapy (1 mg/kg/day), and then the patients were maintained on oral steroid with dosage gradually reduced within variable duration based on disorder recovery and intravenous immunoglobulin (IVIg) referred to 2.0 g/kg for children. Treatments in which the patients had access to were classified as inadequate and adequate treatment. Inadequate treatment included steroid not being tapered off, an insufficient dose of acetazolamide (AZA) <2 mg/kg/day, mycophenolate mofetil (MMF) <500 mg/m^2^/dose, or AZA/MMF treatment duration for less than 6 months, mainly due to the patients’ non-adherence to their prescribed treatment regimen, adverse drug reactions, or personal economic burdens for long-term drug use. Adequate treatment was defined as steroid tapered with or without the usage of AZA ≧ 2 mg/kg/day or MMF ≧ 500 mg/m^2^/dose for more than 6 months. The improvement of clinical outcome was classified as “poor recovery”, “median recovery”, and “good recovery” assigned to the extreme third ends for 1-year assessment, respectively for 0%–33%, 34%–65%, and 66%–100% improvement as indicated in a previous study (20).

### Statistical Analysis

We performed statistical analyses with SPSS V.23.0 (SPSS, Chicago, IL, USA), and the figures were generated with GraphPad Prism 8.0 (GraphPad Software, La Jolla, CA, USA). Continuous variables were expressed as means ± SD or medians with ranges. The χ^2^ or Fisher’s exact test was used to compare the discrete variables, while a Student’s t-test or Mann–Whitney U test was employed to compare the quantitative data of the cohort. We performed an explanatory analysis through the univariate/multivariate Poisson’s and logistic regression approaches. Variables related to a significant change (p < 0.10) at univariate analysis were further analyzed using multivariate Poisson’s or logistic regression. A backward stepwise multivariate logistic regression was used for risk score modeling *via* variable selection with an elimination criterion of p < 0.05. Data were reported with odds ratios (ORs) and 95% CIs. In the risk score model, the risk factors of binary variable were allocated the value of 1 to “yes” for mixed lesions; “≧1” for counts of concomitant autoantibodies and tapering steroid+MMF, tapering steroid+AZA, and tapering steroid only in maintenance therapy; and the value of 0 otherwise. The goodness-of-fit value was calculated by the Pearson/Spearman test. Multiple imputations were used for missing values by SPSS.

Nomogram variables were ranked to generate the model after multivariate logistic regression analysis and risk factor selection based on their p-values <0.05 and further validated in the validation cohort. The use of a nomogram was described as follows: the variable points of an individual patient were measured based on each variable axis of the nomogram. The sum of the points was shown at the total points axis, which reflected the probability of relapse in a 1-year follow-up in the risk axis. We employed the concordance index (C-index) to assess nomogram discrimination; that is, a larger C-index referred to a higher degree of accuracy. Calibration was measured to evaluate the predicted probabilities of the nomogram and assessed through the Hosmer–Lemeshow test. Decision curve analysis (DCA) was performed to measure the clinical net benefit using the nomogram model, and the clinical impact curve (CIC) was used to evaluate the clinical effect of the nomogram. The net reclassification improvement (NRI) and integrated discrimination improvement (IDI) were implemented to evaluate the improvement of the risk difference of the nomogram. Statistical significance was set at p < 0.05.

## Results

### The Demographics of Pediatric Patients With Neuromyelitis Optica Spectrum Disorders

The demographics of patients in the primary cohort are summarized in **Table 1**. A total of 64 pediatric patients with age ≤ 18 years, including 39 with positive serum AQP4 antibody, 12 with positive serum MOG antibody, and 13 with negative serum AQP4/MOG antibody, were assigned into the primary cohort. The majority of patients (60.94%) were female. The most common onset episode was ON+brain/brainstem (32.81%), followed by brain/brainstem only (23.44%), TM+brain/brainstem (17.19%), isolated ON (12.50%), ON+TM (10.93%), and ON+TM+brain/brainstem (3.13%), suggesting that the disease onset of the pediatric patients was preferential to brain/brainstem involvement. Similarly, 46.88% of relapse manifestation was brain/brainstem onset, followed by ON (35.94%) and TM (25.00%). The median pretreatment and discharge EDSS were 3.00 (3.00–6.00) and 2.00 (1.00–3.00), respectively. We found count (≧1) of positive concomitant autoantibody in 29 patients (45.30%), while 18 patients (28.10%) has ≥2 positive concomitant autoantibodies. Twenty-eight patients experienced 35 relapse attacks in 1-year follow-up. The majority of subjects (73.44%) had access to adequate treatment, whereas 26.56% were treated inadequately. CSF white blood count (WBC) count and protein level differed between the children with seropositive AQP4 antibody, seropositive MOG antibody, and seronegative AQP4/MOG antibody (p = 0.023 and 0.021, respectively), while these antibody subgroups did not differ significantly in CSF IgG and IgM, and oligoclonal bands (OCBs) in CSF and serum or in CSF alone, respectively.

**Table 1:** The demographics and clinical characteristics of pediatric NMOSD patients with positive AQP4 antibody, positive MOG antibody, and double-antibody negative.

Feature	AQP4 cohort (n = 39)	MOG cohort (n = 12)	AQP4 and MOG negative (n = 13)	Total cohort (n = 64)	p-Value
**Onset characteristics**					
Age at onset, median (range), year	15 (10–16)	8 (6–14.5)	9 (8–10.5)	11 (9–15.75)	0.003**
Gender					
Male, %	8 (20.50)	9 (75.00)	8 (61.50)	25 (39.06)	0.001**
Female, %	31 (79.50)	3 (25.00)	5 (38.50)	39 (60.94)	
ARR pretreatment, median (range)	0.00 (0.00–1.00)	0.00 (0.00–1.00)	0.00 (0.00–1.00)	0.00 (0.00–1.00)	0.624
Antibody titer at onset, median (range)					
Serum	1:32 (1:10–1:32)	1:32 (1:10–1:100)	0 (0–0)	1:10 (1:10–1:32)	0.001**
CSF	0 (0–1:10)	0 (0–0)	0 (0–0)	0 (0–0)	0.027*
Onset episode, no. (%)					
ON only	8 (20.51)	0 (0)	0 (0)	8 (12.50)	<0.001***
TM only	0 (0)	0 (0)	0 (0)	0 (0)	
Brain/brainstem only	10 (25.64)	5 (41.67)	0 (0)	15 (23.44)	
ON+TM	3 (7.69)	4 (33.33)	0 (0)	7 (10.93)	
ON+brain/brainstem	7 (17.95)	3 (25.00)	11 (84.62)	21 (32.81)	
TM+brain/brainstem	9 (23.08)	0 (0)	2 (15.38)	11 (17.19)	
ON+TM+brain/brainstem	2 (5.13)	0 (0)	0 (0)	2 (3.13)	
Time interval of hospitalization, day, median (range)	14.00 (12.00–17.00)	15.50 (13.00–20.00)	16.00 (10.50–19.00)	15.00 (12.00–17.75)	0.293
Visual disability at onset, n (%)	6 (15.38)	3 (25.00)	4 (30.77)	13 (23.30)	0.342
Baseline EDSS, median (range)	0.00 (0.00–0.00)	0.00 (0.00–1.00)	0.00 (0.00–1.00)	0.00 (0.00–1.00)	0.460
Pretreatment EDSS, median (range)	3.00 (3.00–6.00)	3.00 (2.00–4.00)	7.00 (3.00–8.00)	3.00 (3.00–6.00)	0.004**
Discharge EDSS, median (range)	2.00 (1.00–3.00)	0.50 (0.00–1.00)	3.00 (1.50–4.00)	2.00 (1.00–3.00)	0.005**
Count of concomitant autoantibodies^a^, no. (%)					
≥1	20 (51.30)	3 (25.00)	6 (46.20)	29 (45.30)	0.316
<1	19 (48.70)	9 (75.00)	7 (53.80)	35 (54.70)	
Count of concomitant autoantibodies^a^, no. (%)					
≥2	15 (38.50)	2 (16.70)	1 (7.70)	18 (28.10)	0.070
<2	24 (61.50)	10 (83.30)	12 (92.30)	46 (71.90)	
Serum IgG, mean (range), g/L	17.60 (10.70–24.82)	13.80 (11.37–23.16)	18.60 (11.23–24.55)	17.03 (11.13–23.16)	0.969
Complement C3, median (range), g/L	1.19 (1.05–1.32)	1.33 (1.00–1.59)	1.33 (0.98–1.46)	1.19 (1.05–1.36)	0.383
**Follow-up**					
Number of patients with relapse in 1 year follow-up (%)	14 (35.90)	4 (33.33)	10 (76.90)	28 (43.75)	0.027*
Number of attacks within 1 year follow-up	18	4	13	35	0.022*
Relapse episode, no. (%)					
ON	8 (20.50)	6 (50.00)	9 (69.20)	23 (35.94)	0.004**
TM	12 (30.80)	3 (25.00)	1 (7.70)	16 (25.00)	0.256
Brain/brainstem	17 (43.60)	6 (50.00)	7 (53.80)	30 (46.88)	0.794
**Treatment variables, no. (%)**					
Adequate treatment	32 (82.05)	9 (75.00)	6 (46.15)	47 (73.44)	0.160
With steroid tapering	22 (56.41)	5 (41.70)	6 (46.15)	33 (51.56)	
With steroid tapering+AZA	6 (15.38)	3 (25.00)	0 (0)	9 (14.06)	
With steroid tapering+MMF	4 (10.26)	1 (8.30)	0 (0)	5 (7.81)	
Inadequate treatment	7 (17.95)	3 (25.00)	7 (53.85)	17 (26.56)	
**CSF findings**					
CSF WBC count (×106), median (range)	10 (4–64)	14 (8.5–60)	1 (1–15)	9 (2–45)	0.023*
CSF protein, median (range), g/L	0.30 (0.22–0.41)	0.33 (0.17–0.45)	0.20 (0.16–0.27)	0.26 (0.19–0.39)	0.021*
CSF IgG, median (range), g/L	29.50 (18.00–43.20)	32.20 (17.70–37.60)	20.40 (10.40–32.40)	27.20 (16.00–37.60)	0.376
CSF IgM, median (range), g/L	0.45 (0.20–2.43)	0.91 (0.39–1.15)	0.26 (0.26–0.40)	0.45 (0.26–1.49)	0.183
OCBs in CSF and serum, no. (%)	1 (2.56)	0 (0)	0 (0)	1 (1.56)	0.105
OCBs in CSF alone, no. (%)	1 (2.56)	0 (0)	0 (0)	1 (1.56)	0.105

NMOSD, neuromyelitis optica spectrum disorder; AQP4, aquaporin-4; MOG, myelin oligodendrocyte glycoprotein; ON, optic neuritis; TM, transverse myelitis; ARR, annualized relapse rate; EDSS, Expanded Disability Status Scale; CSF, cerebrospinal fluid; OCBs, oligoclonal bands.

*p < 0.05; **p < 0.01; ***p < 0.001.

a Including antinuclear antibody, extractable nuclear antigen antibody, double-stranded DNA antibody, antineutrophil cytoplasmic antibody, anticardiolipin antibody, Sjögren’s syndrome A antibody, Sjögren’s syndrome B antibody, rheumatoid factor, anti-oipA antibody, thyroglobulin antibody, and thyroid peroxidase antibody.

### The Clinical Characteristics of Patients With/Without Relapse in 1-Year Follow-Up

As indicated in **Table 2**, there were 28 patients who experienced the first relapse and 36 with no relapse in a 1-year follow-up in the primary cohort. The median pretreatment EDSS and discharge EDSS in the children with relapse were higher as compared with that in the children with no relapse (median 5.00 (3.00–8.00) vs. median 3.00 (2.00–5.50), p = 0.003; median 3.00 (1.00–4.00) vs. median 1.00 (0.00–2.50), p = 0.004). Brain/brainstem onset in the children with relapse was more frequent than in the children without relapse (p = 0.001) as well as mixed-lesion onset (p = 0.001). We found that a higher frequency for ≧1 concomitant autoantibodies was observed in the patients with relapse than in the patients without relapse (p = 0.001). In maintenance therapy, adequate therapies were applied in children with no relapse more frequently as compared with the children with relapse (p = 0.004), suggesting that long-term optimal therapeutic management could restrain recurrent onset. Additionally, the clinical characteristics of pediatric patients were compared between the primary cohorts and validation cohorts and listed in **Table 3**.

**Table 2:** Univariate analysis between patients with/without relapse in the primary cohort.

Feature	Relapse (n = 28)	Non-relapse (n = 36)	p-Value
Gender			
Female	16 (57.10)	23 (63.90)	0.583
Male	12 (42.90)	13 (36.10)	
Age at onset, median (range), years	9.50 (9.00–15.00)	13.00 (9.00–16.00)	0.270
Antibody status, no. (%)			
AQP4 antibody positive	14 (50.00)	25 (69.40)	0.114
AQP4 antibody negative	14 (50.00)	11 (30.60)	
ARR pretreatment	0 (0–1)	0 (0–1)	0.945
Visual disability at onset, no. (%)			
Yes	5 (17.90)	8 (22.20)	0.667
No	23 (82.10)	28 (77.80)	
Pretreatment EDSS, median (range)	5.00 (3.00–8.00)	3.00 (2.00–5.50)	0.003**
Discharge EDSS, median (range)	3.00 (1.00–4.00)	1.00 (0.00–2.50)	0.004**
Attack at onset, no. (%)			
ON			
Yes	16 (57.10)	22 (61.10)	0.748
No	12 (42.90)	14 (38.90)	
TM			
Yes	11 (39.30)	9 (25.00)	0.221
No	17 (60.70)	27 (75.00)	
Brain/brainstem			
Yes	27 (96.40)	22 (61.10)	0.001**
No	1 (3.60)	14 (38.90)	
Mixed lesion^#^			
Yes	24 (85.70)	17 (47.20)	0.001**
No	4 (14.30)	19 (52.80)	
Antibody titer, no. (%)			
≥1:32	16 (57.10)	14 (38.90)	0.147
<1:32 or negative	12 (42.90)	22 (61.10)	
Counts of concomitant autoantibodies^§^, no. (%)			
≥1	19 (67.90)	10 (27.80)	0.001**
<1	9 (32.10)	26 (72.20)	
Counts of concomitant autoantibodies^§^, no. (%)			
≥2	8 (28.60)	10 (27.80)	0.944
<2	20 (71.40)	26 (72.20)	
Serum IgG, mean ± SD, g/L	18.49 ± 8.19	17.28 ± 10.00	0.611
Complement C3, median (range), g/L	1.22 (1.01–1.37)	1.19 (1.06–1.35)	0.772
CSF protein, median (range), g/L	0.27 (0.17–0.39)	0.26 (0.19–0.41)	0.944
CSF white cell count (×10^6^), median (range)	14 (1–37)	6 (3.5–61)	0.566
CSF IgG, median (range), g/L	25.35 (16.20–37.55)	29.80 (16.00–37.60)	0.683
CSF IgM, median (range), g/L	0.45 (0.26–1.11)	0.43 (0.20–1.50)	0.921
Acute therapy, no. (%)			
High-dose steroid + IVIg	14 (50.00)	14 (38.90)	0.35
IVIg	4 (14.30)	2 (5.60)	
High-dose steroid	5 (17.90)	12 (33.30)	
Not with high-dose steroid/optimal IVIg	5 (17.90)	8 (22.20)	
Maintenance therapy			
Tapering steroid+MMF	1 (3.60)	4 (11.10)	0.004**
Tapering steroid+AZA	1 (3.60)	8 (22.20)	
Tapering steroid only	13 (46.40)	20 (55.60)	
Inadequate treatment	13 (46.40)	4 (11.10)	
PB B cell count, median (range), %	31.49 (10.00–44.46)	26.88 (21.07–35.14)	0.772
PB NK cell count	13.98 (6.95–19.54)	14.12 (9.63–22.04)	0.449
PB CD4+ cell count	25.77 (18.73–36.51)	26.29 (17.60–32.59)	0.435
PB CD8+ cell count	23.29 (20.75–27.88)	24.77 (17.81–30.20)	0.994
PB CD4+/CD8+	1.19 (0.80–1.54)	1.18 (0.60–1.84)	0.756

AQP4, aquaporin-4; MOG, myelin oligodendrocyte glycoprotein; ON, optic neuritis; TM, transverse myelitis; EDSS, Expanded Disability Status Scale.

*p < 0.05; **p < 0.01; ***p < 0.001.

^§^Including antinuclear antibody, extractable nuclear antigen antibody, double-stranded DNA antibody, antineutrophil cytoplasmic antibody, anticardiolipin antibody, Sjögren’s syndrome A antibody, Sjögren’s syndrome B antibody, rheumatoid factor, anti-oipA antibody, thyroglobulin antibody, and thyroid peroxidase antibody.

^#^Including ON+TM, ON+cerebrum/brainstem, TM+cerebrum/brainstem, and ON+TM+cerebrum/brainstem.

**Table 3:** The demographics and clinical characteristics of pediatric patients in primary cohort and validation cohort.

Variables	Primary cohort	Validation cohort	p-Value
Number of patients	64	35	─
Antibody category, no. (%)			
AQP4 antibody positive	39 (60.90)	12 (34.30)	0.011*
AQP4 antibody negative	25 (39.10)	23 (65.70)	
Number of patients with relapse in 1 year following up, no. (%)	28 (43.80)	12 (34.30)	0.361
Number of attacks within 1 year following up, median (range)	0 (0–1)	0 (0–1)	0.221
ARR pretreatment, median (range)	0.00 (0.00–1.00)	0.00 (0.00–0.50)	0.572
Time interval from disease onset to first relapse, median (range), m	1.00 (0.00–6.75)	0.00 (0.00–7.00)	0.433
Age at onset, median (range), year	11 (9–15.75)	7 (6–11)	<0.001***
Gender			
Male, no. (%)	25 (39.10)	13 (37.10)	0.851
Female, no. (%)	39 (60.90)	22 (62.90)	
Serum antibody titer at onset, median (range)			
AQP4 antibody	1:10 (0–1:32)	0 (0–1:10)	0.179
MOG antibody	0 (0–1:10)	0 (0–1:10)	0.056
CSF antibody titer at onset, median (range)			
AQP4 antibody	0 (0–0)	0 (0–0)	0.055
MOG antibody	0 (0–0)	0 (0–0)	0.176
Onset episode, no. (%)			
ON	38 (59.40)	23 (65.70)	0.535
TM	20 (31.30)	17 (48.60)	0.089
Brain/brainstem	49 (76.60)	21 (60.00)	0.107
Baseline EDSS, median (range)	0.00 (0.00–1.00)	0.00 (0.00–1.00)	0.893
Pretreatment EDSS, median (range)	3.00 (3.00–6.00)	4.00 (3.00–6.00)	0.141
Discharge EDSS, median (range)	2.00 (1.00–3.00)	1.00 (1.00–2.00)	0.284
Count of concomitant autoantibodies^a^, no. (%)			
≥1	29 (45.30)	15 (42.90)	0.814
<1	35 (54.70)	20 (57.10)	
Count of concomitant autoantibodies^a^, no. (%)			
≥2	18 (28.10)	6 (17.10)	0.223
<2	46 (71.90)	29 (82.90)	
Treatment variables, no. (%)			
Adequate treatment	47 (73.40)	29 (82.90)	0.714
Inadequate treatment	17 (26.60)	6 (17.10)	

AQP4, aquaporin-4; MOG, myelin oligodendrocyte glycoprotein; ON, optic neuritis; TM, transverse myelitis; ARR, annualized relapse rate; EDSS, Expanded Disability Status Scale.

a Including antinuclear antibody, extractable nuclear antigen antibody, double-stranded DNA antibody, antineutrophil cytoplasmic antibody, anticardiolipin antibody, Sjögren’s syndrome A antibody, Sjögren’s syndrome B antibody, rheumatoid factor, anti-oipA antibody, thyroglobulin antibody, and thyroid peroxidase antibody.

### Establishment of Signature Associated With Risk Factors for Relapse Prediction in 1-Year Follow-Up

Using univariate Poisson’s regression (**Table 4**), we identified tapering steroid+AZA and tapering steroid only with a decreased OR compared with inadequate treatment for counts of relapsed attacks in a 1-year follow-up (p = 0.019 and 0.003, respectively). Pretreatment EDSS, discharge EDSS, brain/brainstem, mixed lesion, and counts (≧1) of concomitant autoantibodies were associated with an increased OR of counts of relapsed attacks (p = 0.002, 0.003, 0.016, 0.002, and 0.005, respectively). No associations were found between counts of relapsed attacks and gender, antibody status, age, ON and TM onset, serum antibody titer, counts (≧2) of concomitant autoantibodies, CSF protein level, CSF white cell count, CSF IgG and IgM level, acute therapy, and peripheral blood lymphocyte percentage (e.g., B cell, NK cell, and CD4+ T cell). Using multivariate Poisson’s regression, we further defined that mixed-lesion onset and counts (≧1) of concomitant autoantibodies were independently associated with an increased OR of counts of relapsed attacks (p = 0.007 and 0.019), while maintenance therapy for steroid tapered only had a decreased OR for counts of relapsed attack (p = 0.008).

**Table 4:** Risk factors of relapse attacks using univariate and multivariate Poisson’s regression in 1-year follow-up.

Variable	Univariate analysis		Multivariate analysis	
	OR (95% CI)	p-Value	OR (95% CI)	p-Value
Gender				
Female	0.962 (0.523–1.768)	0.900	─	─
Male	Ref		─	─
Age	0.971 (0.900–1.047)	0.445	─	─
Antibody status				
AQP4 antibody positive	0.679 (0.372–1.239)	0.207	─	─
AQP4 antibody negative	Ref		─	─
ARR pretreatment	1.022 (0.520–2.008)	0.949	─	─
Visual disability at onset				
Yes	0.981 (0.431–2.234)	0.963	─	─
No	Ref		─	─
Pretreatment EDSS	1.178 (1.062–1.306)	0.002**	─	─
Discharge EDSS	1.247 (1.077–1.443)	0.003**	─	─
Attack at onset				
ON				
Yes	1.026 (0.557–1.892)	0.934	─	─
No	Ref		─	─
TM				
Yes	1.467 (0.801–2.684)	0.214	─	─
No	Ref		─	─
Brain/brainstem				
Yes	10.408 (1.534–70.626)	0.016*	─	─
No	Ref		─	─
Mixed lesion^#^				
Yes	4.348 (1.702–11.106)	0.002**	3.398 (1.390–8.306)	0.007**
No	Ref		Ref	
Serum AQP4/MOG antibody titer				
≥1:32	1.346 (0.724–2.503)	0.348	─	─
<1:32 or negative	Ref		─	─
Counts of concomitant autoantibodies^§^				
≥1	2.633 (1.336–5.191)	0.005**	1.967 (1.120–3.456)	0.019*
<1	Ref		Ref	
Counts of concomitant autoantibodies^§^				
≥2	1.171 (0.596–2.301)	0.646	─	─
<2	Ref		─	─
CSF protein level	0.667 (0.163–2.716)	0.571	─	─
CSF white cell count	0.997 (0.990–1.003)	0.327	─	─
CSF IgG	0.998 (0.991–1.006)	0.674	─	─
CSF IgM	0.981 (0.910–1.057)	0.611	─	─
Acute therapy				
High-dose steroid + IVIg	1.315 (0.561–3.085)	0.528	─	─
IVIg	1.806 (0.666–4.892)	0.245	─	─
High-dose steroid	0.892 (0.299–2.661)	0.838	─	─
Not with high-dose steroid/optimal IVIg	Ref			
Maintenance therapy				
Tapering steroid+MMF	0.189 (0.032–1.123)	0.067	─	─
Tapering steroid+AZA	0.105 (0.016–0.685)	0.019*	─	─
Tapering steroid only	0.429 (0.245–0.752)	0.003**	0.503 (0.302–0.837)	0.008**
Inadequate treatment	Ref		Ref	
PB B cell count	1.005 (0.982–1.029)	0.663	─	─
PB NK cell count	0.980 (0.945–1.016)	0.265	─	─
PB CD4+ cell count	1.008 (0.984–1.034)	0.510	─	─
PB CD8+ cell count	1.018 (0.982–1.054)	0.338	─	─
PB CD4+/CD8+	0.938 (0.638–1.381)	0.747	─	─

NMOSD, neuromyelitis optica spectrum disorder; AQP4, aquaporin-4; MOG, myelin oligodendrocyte glycoprotein; ON, optic neuritis; TM, transverse myelitis; EDSS, Expanded Disability Status Scale.

*p < 0.05; **p < 0.01; ***p < 0.001.

^§^Including antinuclear antibody, extractable nuclear antigen antibody, double-stranded DNA antibody, antineutrophil cytoplasmic antibody, anticardiolipin antibody, Sjögren’s syndrome A antibody, Sjögren’s syndrome B antibody, rheumatoid factor, anti-oipA antibody, thyroglobulin antibody, and thyroid peroxidase antibody.

^#^Including ON+TM, ON+cerebrum/brainstem, TM+cerebrum/brainstem, and ON+TM+cerebrum/brainstem.

Through univariate logistic regression (**Table 5**), we identified maintenance therapy including oral steroid with or without MMF/AZA with a decreased OR of the relapse compared with inadequate treatment (p = 0.041, 0.007, and 0.017). Pretreatment EDSS, discharge EDSS, brain/brainstem onset, mixed lesion, and counts (≧1) of concomitant autoantibodies were associated with an increased OR of the relapse in a 1-year follow-up (p = 0.004, 0.005, 0.008, 0.003, and 0.002, respectively). Multivariate logistic regression further demonstrated that discharge EDSS, mixed lesion, and counts (≧1) of concomitant autoantibodies were independently associated with an increased OR of the occurrence of relapse (p = 0.017, 0.010, and 0.015, respectively), whereas maintenance therapy was associated with a decreased OR of the occurrence of relapse (p = 0.009, 0.045, and 0.025, respectively).

**Table 5:** Risk factors of relapse using univariate and multivariate logistic regressions in 1-year follow-up.

Variable	Univariate analysis		Multivariate analysis (model 1)		Multivariate analysis (model 2)	
	OR (95% CI)	p-Value	OR (95% CI)	p-Value	OR (95% CI)	p-Value
Gender						
Female	0.754 (0.274–2.072)	0.584	─	─	─	─
Male	Ref		─	─	─	─
Age	0.932 (0.824–1.055)	0.266	─	─	─	─
Antibody status						
AQP4 antibody positive	0.440 (0.158–1.227)	0.117	─	─	─	─
AQP4 antibody negative	Ref		─	─	─	─
ARR Pretreatment	1.040 (0.347–3.116)	0.944	─	─	─	─
Visual disability at onset						
Yes	0.761 (0.219–2.645)	0.667	─	─	─	─
No	Ref		─	─	─	─
Pretreatment EDSS	1.437 (1.124–1.837)	0.004**	─	─	─	─
Discharge EDSS	1.698 (1.173–2.456)	0.005**	2.100 (1.143–3.859)	0.017**	─	─
Attack at onset						
ON						
Yes	0.848 (0.311–2.317)	0.749	─	─	─	─
No	Ref		─	─	─	─
TM						
Yes	1.941 (0.667–5.658)	0.224	─	─	─	─
No	Ref		─	─	─	─
Brain/brainstem						
Yes	17.182 (2.093–141.070)	0.008**	─	─	─	─
No	Ref		─	─	─	─
Mixed lesion^#^						
Yes	6.706 (1.932–23.276)	0.003**	11.348 (1.773–72.625)	0.010*	─	─
No	Ref		Ref		─	─
Serum AQP4/MOG antibody titer						
≥1:32	2.095 (0.767–5.722)	0.149	─	─	─	─
<1:32 or negative	Ref		─	─	─	─
Counts of concomitant autoantibodies^§^						
≥1	5.489 (1.869–16.122)	0.002**	7.733 (1.500–39.873)	0.015*	─	─
<1	Ref		Ref		─	─
Counts of concomitant autoantibodies^§^						
≥2	1.040 (0.347–3.116)	0.944	─	─	─	─
<2	Ref		─	─	─	─
CSF protein	0.875 (0.116–6.623)	0.897	─	─	─	─
CSF white cell count	0.995 (0.984–1.007)	0.428	─	─	─	─
CSF IgG	1.001 (0.988–1.012)	0.935	─	─	─	─
CSF IgM	0.984 (0.876–1.105)	0.785	─	─	─	─
Acute therapy						
High-dose steroid + IVIg	1.600 (0.409–6.114)	0.492	─	─	─	─
IVIg	3.200 (0.419–24.417)	0.262	─	─	─	─
High-dose steroid	0.667 (0.145–3.075)	0.603	─	─	─	─
Not with high-dose steroid/optimal IVIg	Ref		─	─	─	─
Maintenance therapy						
Tapering steroid+MMF	0.077 (0.007–0.901)	0.041*	0.017 (0.001–0.369)	0.009**	─	─
Tapering steroid+AZA	0.038 (0.004–0.408)	0.007**	0.060 (0.004–0.939)	0.045*	─	─
Tapering steroid only	0.200 (0.053–0.749)	0.017*	0.098 (0.013–0.749)	0.025*	─	─
Inadequate treatment	Ref		Ref			
PB B cell count	1.003 (0.964–1.042)	0.899	─	─	─	─
PB NK cell count	0.983 (0.938–1.031)	0.484	─	─	─	─
PB CD4+ cell count	1.019 (0.971–1.068)	0.443	─	─	─	─
PB CD8+ cell count	1.018 (0.955–1.086)	0.583	─	─	─	─
PB CD4+/CD8+	1.011 (0.483–2.116)	0.977	─	─	─	─
Risk score	2.576 (1.640–4.044)	<0.001**	─	─	2.560 (1.060–6.183)	0.037*

NMOSD, neuromyelitis optica spectrum disorder; AQP4, aquaporin-4; MOG, myelin oligodendrocyte glycoprotein; ON, optic neuritis; TM, transverse myelitis; EDSS, Expanded Disability Status Scale; IVIg, intravenous immunoglobulin; MMF, mycophenolate mofetil; AZA, azathioprine.

*p < 0.05; **p < 0.01; ***p < 0.001.

^§^Including antinuclear antibody, extractable nuclear antigen antibody, double-stranded DNA antibody, antineutrophil cytoplasmic antibody, anticardiolipin antibody, Sjögren’s syndrome A antibody, Sjögren’s syndrome B antibody, rheumatoid factor, anti-oipA antibody, thyroglobulin antibody, and thyroid peroxidase antibody.

^#^Including ON+TM, ON+cerebrum/brainstem, TM+cerebrum/brainstem, and ON+TM+cerebrum/brainstem.

Next, the expressive profile of these four independent factors from multivariate logistic regression was computed, and the risk model of relapse was calculated as (0.74 × expression value of discharge EDSS) + (2.43 × expression value of mixed lesion) + (2.05 × expression value of counts (≧1) of concomitant autoantibodies) + (0 × expression value of inadequate treatment)/(−4.07 × expression value of steroid taper+MMF)/(−2.81 × expression value of steroid taper+AZA)/(−2.32 × expression value of steroid taper only). To identify the risk score model as an indicator for relapse, the univariate logistic regression was conducted to verify the risk score presenting an increased OR of the occurrence of relapse (p < 0.001), and multivariate logistic regression revealed that the risk score served as a unique risk factor with an increased OR of the occurrence of relapse in model 2 (p = 0.037) (**Table 5**).

**Figure 1:**
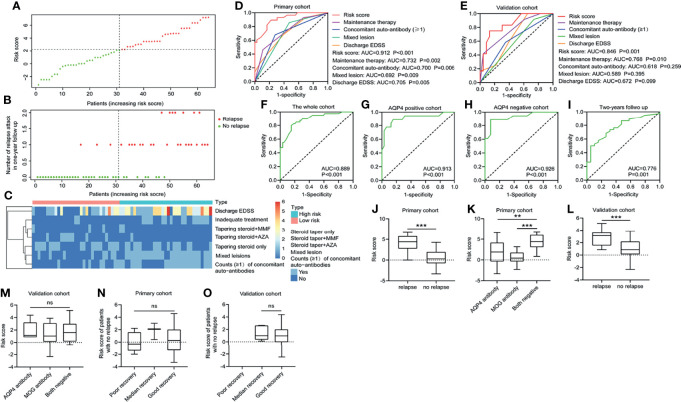
Predictive value of the risk score model. **(A, B)** The 4-independent-factors signature risk score distribution with the relapse status of pediatric patients. The colorgram of 4-independent-factor expression profiles of high- and low-risk groups. The vertical line represents the median cutoff dividing patients into low- and high-risk groups. **(C)** The expression of cluster heatmap for the four risk factors in the primary cohort. ROC for recurrence in 1-year follow-up in the primary cohort **(D)** and validation cohort **(E)** using risk score compared with other indicators. ROC for recurrence in 1-year follow-up in the whole cohort **(F)**, AQP4 antibody-positive cohort **(G)**, AQP4 antibody-negative cohort **(H)**, and the cohort with 2-year follow-up **(I)**. **(J)** Risk score distribution in the primary cohort with or without relapse in 1-year follow-up. **(K)** Risk score distribution of different antibody types in the primary cohort. **(L)** Risk score distribution in the validation cohort with/without relapse in 1-year follow-up. **(M)** Risk score distribution of different antibody subsets in the validation cohort. For patients without relapse in 1-year follow-up, risk score distribution was detected in the primary cohort **(N)** and the validation cohort **(O)** according to different clinical outcomes at 1 year. EDSS, Expanded Disability Status Scale; AQP4, aquaporin-4; MOG, myelin oligodendrocyte glycoprotein; AZA, acetazolamide; MMF, mycophenolate mofetil; ns, not statistically significant; ROC, receiver operating characteristic. **p < 0.01 and ***p < 0.001.

Then the risk score was calculated for each patient in the primary cohort and divided all patients into high- and low-risk groups based on the cutoff value of the receiver operating characteristic (ROC) curve (**Figure 1A**). It was found in **Figure 1B** that most of the patients with relapse belonged to the high-risk group. **Figure 1C** shows the cluster heatmap profile of discharge EDSS, maintenance therapy, mixed lesion, and counts (≧1) of concomitant autoantibodies in the primary cohort. The ROC curve revealed that the risk score model could predict relapsed attacks in a 1-year follow-up in the primary cohort more effectively as compared with discharge EDSS, mixed lesion, counts (≧1) of concomitant autoantibodies, and maintenance therapy (AUC of 0.912 vs. AUC of 0.705, 0.692, 0.700, and 0.732 respectively) (**Figure 1D**). This phenomenon was also indicated in the validation cohort (AUC of 0.846 vs. AUC of 0.672, 0.589, 0.618, and 0.768) (**Figure 1E**). The AUC of the risk score model was 0.889 in the whole cohort (combination of the primary cohort and validation cohort) (**Figure 1F**). The whole cohort was further divided into AQP4 antibody-positive and AQP4 antibody-negative cohorts. The AUC of the risk score model was 0.913 in the AQP4 antibody-positive cohort (**Figure 1G**) and 0.926 in the AQP4 antibody-negative cohort (**Figure 1H**), more than in the cohort with 2-year follow-up (AUC of 0.776) (**Figure 1I**). Additionally, we found that in the primary cohort, the children with relapse usually had higher risk scores than the children with no relapse (p < 0.001) (**Figure 1J**), and this was also verified in the validation cohort (p < 0.001) (**Figure 1L**). In the primary cohort, the higher risk score was found in the patients with negative AQP4/MOG antibody compared with AQP4 antibody-positive patients and MOG antibody-positive patients, respectively (p < 0.010; p < 0.001) (**Figure 1K**), but this finding was not observed in the validation cohort (p > 0.05) (**Figure 1M**). This may be caused by the patient heterogeneity between the primary and validation cohorts. For the patient with no relapse in the primary cohort (**Figure 1N**) and the validation cohort (**Figure 1O**), there was no association between individual risk score and recovery grade at 1 year.

### The Nomogram Model of Relapse in 1-Year Follow-Up

**Figure 2:**
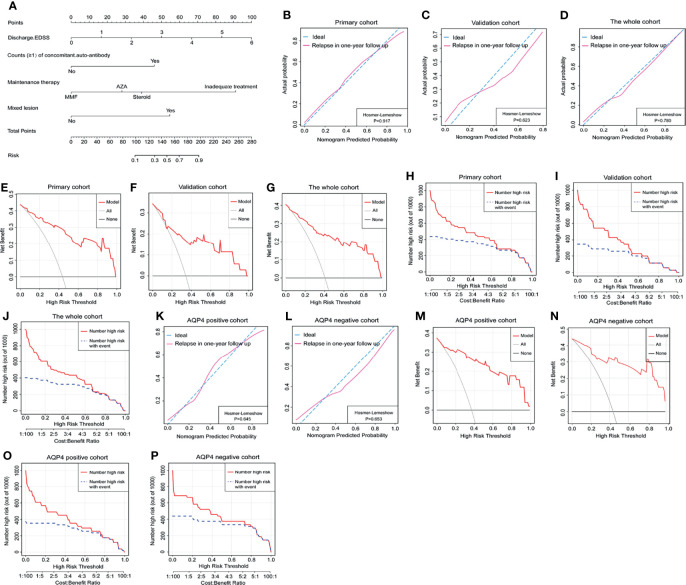
The nomogram model predicted relapse attacks in 1-year follow-up. **(A)** Nomogram was conveyed to predict the probability of recurrence in 1-year follow-up using 4 independent factors. The calibration curve was a plot to predict the probability of recurrence in 1-year follow-up in the primary cohort **(B)**, the validation cohort **(C)**, and the whole cohort **(D)**. Decision curve analysis of relapse in 1-year follow-up in the primary cohort **(E)**, the validation cohort **(F)**, and the whole cohort **(G)**. The clinical impact curve of nomogram for relapse event in 1-year follow-up in the primary cohort **(H)**, the validation cohort **(I)**, and the whole cohort **(J)**. **(K, L)** The calibration curve was used to predict the probability of relapse in AQP4 antibody-positive cohort and AQP4 antibody-negative cohort from the whole cohort. **(M, N)** Decision curve analysis of relapse in 1-year follow-up in AQP4 antibody-positive cohort and AQP4 antibody-negative cohort from the whole cohort. **(O, P)** Clinical impact curve for relapse in 1-year follow-up in AQP4 antibody-positive cohort and AQP4 antibody-negative cohort from the whole cohort. EDSS, Expanded Disability Status Scale; AZA, acetazolamide; MMF, mycophenolate mofetil; AQP4, aquaporin-4.

We further created a predictive model for the occurrence of relapses using a nomogram (**Figure 2A**). The score of each risk factor can be detected by points scale located at the top of the nomogram. The C-index of the nomogram was 0.93 in the primary cohort, 0.91 in the validation cohort, and 0.92 in the whole cohort. The calibration curve exhibited accurate agreement between the predicted and actual probabilities of relapse in the primary cohort, validation cohort, and whole cohort (Hosmer–Lemeshow p = 0.917, 0.623, and 0.780) (**Figures 2B–D**). According to AQP4 antibody status, AQP4 antibody-positive cohort and AQP4 antibody-negative cohort showed greater matching for relapse possibility (Hosmer–Lemeshow p = 0.645 and 0.653) (**Figures 2K, L**). The DCA indicated that the nomogram had good net benefits for the identification of relapse in a 1-year follow-up in the primary cohort, validation cohort, and whole cohort (**Figures 2E–G**), and likewise, the CIC represented the better predictive value of this nomogram for relapse attacks (**Figures 2H–J**). These findings were also observed in AQP4 antibody-positive cohort and AQP4 antibody-negative cohort (**Figures 2M–P**).

### Comparison of the Prediction Performance of Our Nomogram With Existing Risk Factors

**Figure 3:**
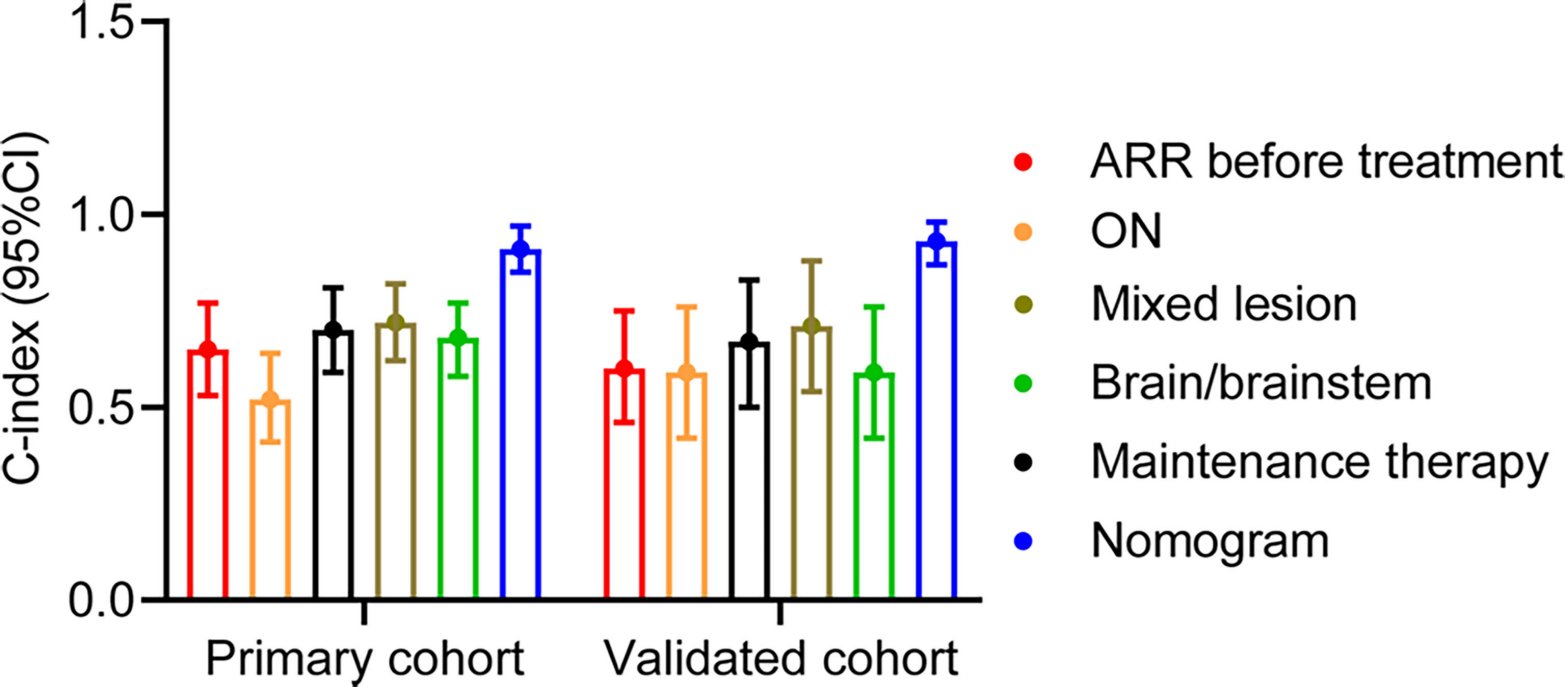
Comparison of the C-indexes between nomogram and other risk factors. ARR, annual relapse rate.

To further evaluate the predictive performance of our nomogram for relapse in the 1-year follow-up, we compared the C-index of our nomogram with other predictive factors reported by previous studies (13, 19, 21). Importantly, our nomogram could efficiently discriminate the patients with relapse from those with no relapse, compared with other factors, like ARR before treatment, ON, and mixed lesion (**Figure 3**). Moreover, in the primary and validation cohorts, our nomogram showed a significant improvement in risk difference using IDI and NRI (continuous) than other reported factors (**Table 6**). Compared with other three-risk-factor models, our nomogram also allowed a significant improvement of risk prediction evaluated by IDI and NRI (continuous) for the primary cohort (all p < 0.05) but did not substantially improve the accuracy in the validation cohort except model C using IDI (p = 0.004) and NRI (continuous) (p < 0.001) (**Table 6**).

**Table 6:** Comparison of nomogram with other predictive factors of relapse.

Nomogram vs.	Primary cohort				Validation cohort			
	IDI (95% CI)	p-Value	NRI (continuous) (95% CI)	p-Value	IDI (95% CI)	p-Value	NRI (continuous) (95% CI)	p-Value
ARR before treatment	0.52 (0.39–0.65)	<0.001***	1.33 (0.96–1.69)	<0.001***	0.38 (0.21–0.54)	<0.001***	0.89 (0.27–1.51)	0.004**
ON	0.52 (0.39–0.65)	<0.001***	1.31 (0.94–1.68)	<0.001***	0.32 (0.11–0.52)	0.003**	1.07 (0.47–1.66)	<0.001***
Maintenance therapy	0.32 (0.20–0.44)	<0.001***	1.19 (0.81–1.59)	<0.001***	0.22 (0.07–0.38)	0.005**	0.73 (0.08–1.38)	0.028*
Mixed lesion	0.36 (0.24–0.48)	<0.001***	1.14 (0.74–1.54)	<0.001***	0.29 (0.13–0.46)	<0.001***	1.07 (0.47–1.66)	<0.001***
Brain/brainstem symptom	0.35 (0.22–0.48)	<0.001***	0.96 (0.53–1.39)	<0.001***	0.39 (0.20–0.57)	<0.001***	0.99 (0.37–1.59)	0.002**
Three-risk-factor model A^α^	0.07 (0.01–0.14)	0.033*	0.80 (0.35–1.25)	<0.001***	0.03 (−0.02 to 0.08)	0.296	0.47 (−0.21 to 1.15)	0.175
Three-risk-factor model B^β^	0.08 (0.01–0.15)	0.024*	0.75 (0.29–1.20)	0.001**	0.08 (−0.03 to 0.19)	0.165	0.55 (−0.12 to 1.22)	0.105
Three-risk-factor model C^γ^	0.13 (0.03–0.22)	0.008**	1.00 (0.58–1.41)	<0.001***	0.19 (0.06–0.31)	0.004**	1.07 (0.45–1.65)	<0.001***
Three-risk-factor model D^δ^	0.08 (0.01–0.16)	0.030*	0.83 (0.41–1.24)	<0.001***	0.04 (−0.02–0.10)	0.232	0.62 (0.04–1.21)	0.037*

EDSS, Expanded Disability Status Scale.

*p < 0.05; **p < 0.01; ***p < 0.001.

^α^Including EDSS+maintenance therapy+mixed lesion.

^β^Including counts (≧1) of concomitant autoantibodies+maintenance therapy+mixed lesion.

^γ^Including EDSS+mixed lesion+counts (≧1) of concomitant autoantibodies.

^δ^Including EDSS+maintenance therapy+counts (≧1) of concomitant autoantibodies.

### Risk Factors of Relapse Presentation in 1-Year Follow-Up

To define whether these risk factors above had predictive value for the clinical manifestation of relapse, univariate logistic regression was conducted to indicate age, positive AQP4 antibody, and tapering steroid only as being with a decreased OR for ON recurrence in a 1-year follow-up (p = 0.015, 0.002, and 0.047, respectively), but mixed lesion was associated with an increased OR of ON recurrence (p = 0.026) (**Table S1**). Using multivariate logistic regression, we defined that the AQP4 antibody was independently associated with a decreased OR of ON recurrence (p = 0.028). As shown in **Table S2**, TM onset was associated with an increased OR of TM recurrence (p = 0.014). In **Table S3**, pretreatment EDSS and brain/brainstem onset were associated with an increased OR of the recurrence of brain/brainstem onset (p = 0.009 and 0.022), while high-dose steroid usage at acute episode was associated with a decreased OR (p = 0.019), and pretreatment EDSS was also associated with an increased OR of mixed-lesion relapse (p = 0.011) (**Table S4**).

### Relapse Prediction in 2-Year Follow-Up Using Nomogram Model

**Figure 4:**
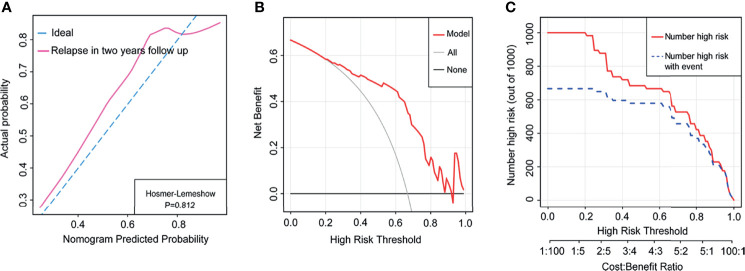
The nomogram model predicted relapse in the whole cohort with 2-year follow-up. **(A)** The calibration curve indicated the probability of relapse in the cohort with 2-year follow-up. **(B)** Decision curve analysis of relapse in the cohort with 2-year follow-up. **(C)** Clinical impact curve of relapse in the cohort with 2-year follow-up.

A total of 57 children, which consisted of 40 from the primary cohort and 17 from the validation cohort, were recruited into the cohort with a 2-year follow-up. The clinical characteristics of the cohort are indicated in **Table S5**. We further assessed the potential of our nomogram predicting relapse possibility in this cohort. The C-index of this nomogram was 0.83 in the cohort. The calibration curve exhibited better agreement in the probability of relapse (Hosmer–Lemeshow p = 0.812) (**Figure 4A**). The DCA indicated that our nomogram presented net benefits for the identification of relapse risk in 2-year follow-up as well (**Figure 4B**), and the CIC represented a notable predictive value of our nomogram in the occurrence of relapse (**Figure 4C**).

## Discussion

Through a series of studies, clinical phenotype, outcome, and treatment strategies of the children with AQP4 antibody-positive NMOSDs were well recognized (11, 22). Interestingly, some pediatric patients with MOG antibody-positive NMOSDs have clinical overlapped manifestations with AQP4 antibody-positive NMOSDs (23), despite the distinct pathology evidence characteristic of severe demyelination without the astrocyte loss usually recognized in AQP4 antibody-positive NMOSD (24). To date, there have been multiple studies evaluating clinical features, treatment response, and relapse possibility among adult patients with NMOSDs (25–27); however, few data were concerned with these characteristics in pediatric patients, especially, Chinese children. Our study investigated the clinical and laboratory data of Chinese pediatric NMOSDs with different antibody subsets, in which we systematically explored the signature of risk factors predicting relapsed attack and generated a nomogram model.

We initially investigated the clinical features of the children with different antibodies. We observed that the children with seropositive AQP4 antibody tended to be older than those with seropositive MOG antibody or seronegative AQP4-/MOG antibody, and a higher female:male ratio in AQP4 antibody-positive pediatric patients was not found in the children with positive MOG antibody or negative AQP4-/MOG antibody. A similar difference in the age and female:male ratio was observed in previous studies as well (6, 28, 29). We also found a difference in the spatial distribution of onset lesions in the children with different antibody types. Brain/brainstem onset was present in 25.64% of AQP4 antibody-positive patients and in 41.67% of MOG antibody-positive patients. Likewise, AQP4/MOG antibody-seronegative patients had a predominant presentation of brain/brainstem onset plus ON. It was speculated that brain/brainstem onset was more likely to be a prominent and distinct feature for pediatric NMOSDs, and this finding was partly supported by previous studies that found that approximately 72% AQP4 antibody-positive pediatric patients had abnormal brain MRI at onset (13), and brain/brainstem syndrome accounted for nearly 40% of the children with AQP4 antibody-positive NMOSDs (30). Furthermore, our study revealed that brain/brainstem onset was more likely to be present again as the sequential attack as compared with other clinical manifestations, and this phenomenon was also found in MOG antibody-mediated disease (31, 32). Interestingly, we found that two children were tested to be both MOG and AQP4 antibody seropositive in the excluded group, and this phenomenon was likely to be influenced by sampling time, sample storage, test conditions, etc.

Next, we found that mixed lesion, counts (≧1) of concomitant autoantibodies and maintenance therapy, as the independent relapse risk factors, could efficaciously predict the occurrence of relapse and even relapse frequency in the 1-year follow-up, whereas discharge EDSS acted as an independent risk factor only for relapse occurrence, not associated with relapse count. This may be caused by the nature distinction between Poisson’s and logistic regression. With the use of these four risk factors, a risk score model was constructed to divide the children into high- and low-risk groups based on ROC cutoff value. Compared with individual factors, this risk score model exhibited better performance to differentiate the children with relapse in the cohort. Actually, several of these 4 factors have been reported to be associated with clinical relapse; for example, maintenance therapies like AZA, MMF, and rituximab treatment have been demonstrated to decrease the risk of relapse and disability for pediatric patients with NMOSDs (6, 12, 19, 33), and the increase in the risk of relapse was observed frequently in the patients starting with ADEM plus ON/TM (mixed lesion) (34).

We further provided a nomogram to predict the probability of relapsed attack. A nomogram’s performance is evaluated in terms of discrimination and calibration, and it is important to consider both when making a clinical decision (35, 36). Through our nomogram generated from the primary cohort, the calibration curve indicated accurate agreement not only in the primary cohort, the validation cohort, and the whole cohort but also in the AQP4 antibody-positive cohort and AQP4 antibody-negative cohort. The C-index of the nomogram exhibited useful discrimination, and the DCA also indicated better net benefits for the nomogram in all the cohorts. Importantly, in comparison with other predictive factors, the nomogram exhibited better risk discrimination and prediction assessed by C-index, NRI, and IDI. The better performance of those indicators guaranteed the accuracy and reliability of our nomogram prediction performance.

Another interesting finding from our study was to identify several independent factors predicting clinical presentation at the second attack. A previous study of MOG antibody-positive NMOSD patients with an age range of 6 to 70 indicated that in 72% of patients initially presenting with isolated ON, the sequential attack was isolated ON again, similar to patients with TM onset (31). These findings suggest that the initial presentation had a higher predictive value for the clinical presentation of the second attack. Additionally, in the AQP4 antibody-positive children, younger patients were more likely to relapse with brain/brainstem onset than older patients (14), and this was clinically distinct from adult patients in that ON attack had more frequency at sequential attack (37, 38). Also, our study demonstrated that TM onset was a potential predictor for TM recurrence at the second attack, and this phenomenon was observed in brain/brainstem onset as well but not found in the children with ON and mixed-lesion onset.

Our study has several limitations. A major limitation is the shorter follow-up duration in the limited sample size to assess the impact of clinical features on the occurrence of relapse. Secondly, there existed a difference in the percentage of AQP4 antibody-positive patients between the primary cohort and validation cohort, perhaps due to the hospitalized patient composition in the different health facilities. Especially, the proportion of AQP4 antibody-positive patients in the whole cohort was higher than in other studies, and this might be a consequence of selection due to a retrospective study. Alternatively, AQP4- and MOG antibody-seronegative NMOSD was more likely to be a heterogeneous disorder entity consisting of multiple unknown pathogeneses affecting treatment responsiveness and relapse risk. Lastly, not all patients were systematically managed with possible biases in treatment initiation. Only two children were administrated with rituximab infusion in the recruited cases in the primary cohort, and their follow-up duration was less than 1 year (data not shown). Therefore, we excluded these two cases from our cohort study. A larger sample size of cases with longer follow-up is warranted to investigate the anti-relapse efficacy of rituximab treatment on Chinese pediatric NMOSDs in our future study.

In conclusion, pediatric NMOSD is a CNS inflammatory condition mediated by different agent autoimmunity, and our study identified a risk model for assessing the relapse risk of Chinese pediatric NMOSDs. Future prospective studies with a larger sample size and longer follow-up are therefore warranted to confirm and extend our findings.

## Data Availability Statement

The original contributions presented in the study are included in the article/**Supplementary Material**. Further inquiries can be directed to the corresponding authors.

## Ethics Statement

The studies involving human participants were reviewed and approved by the ethics committee of Qilu Hospital, Cheeloo College of Medicine, Shandong University. Written informed consent to participate in this study was provided by the participants’ legal guardian/next of kin.

## Author Contributions

XL and YW conceived the study, supervised the work, and participated in its design and coordination. SZ and SQ collected the data and organized the statistical data. SZ drafted the manuscript. HL, RZ, and MW assisted in collecting the data. TH assisted in the statistics and organizing the data. All authors read and approved the final version of the manuscript.

## Funding

This work was supported by grants from the National Natural Science Foundation of China (Nos. 81873786, 81601020, and 31671468), the Natural Science Foundation of Shandong Province, China (No. ZR2016HP04, No. ZR2019MH062), the Academic Promotion Program of Shandong First Medical University (2019QL024), and China Postdoctoral Science Foundation (2021M691227).

## Conflict of Interest

The authors declare that the research was conducted in the absence of any commercial or financial relationships that could be construed as a potential conflict of interest.

## Publisher’s Note

All claims expressed in this article are solely those of the authors and do not necessarily represent those of their affiliated organizations, or those of the publisher, the editors and the reviewers. Any product that may be evaluated in this article, or claim that may be made by its manufacturer, is not guaranteed or endorsed by the publisher.
